# CircTHBS1 targeting miR-211/CCND2 pathway to promote cell proliferation and migration potential in primary cystitis glandularis cells

**DOI:** 10.1042/BSR20201164

**Published:** 2021-08-12

**Authors:** Yue Ma, Zhengfei Shan, Ying Liu, Honggang Shao, Yupeng Xin, Kai He, Shichun Jiang, Yaodong Wang

**Affiliations:** 1 Department of Urology, Mianyang Central Hospital, Mianyang 621000, Sichuan Province, China; 2 Department of Organ Transplantation and Urology, The Affiliated Yantai Yuhuangding Hospital of Qingdao University, Yantai 264000, Shandong Province, China; 3 Department of Oncology, The Affiliated Yantai Yuhuangding Hospital of Qingdao University, Yantai 264000, Shandong Province, China

**Keywords:** CCND2, ceRNA, CircTHBS1, Cystitis glandularis, miR-211

## Abstract

The pathogenesis of cystitis glandular (CG) is unclear, but it is generally considered to be a neoplastic lesion of urothelial hyperplasia formed by long-term chronic stimulation. There is growing evidence that circRNAs play important roles in a variety of cellular processes. However, there are few reports on the role and molecular mechanism of circRNA in CG. In the present study, we first isolated primary cells from CG tissues and adjacent normal tissues. Further experiments showed that CircTHBS1 was up-regulated in primary CG cells (pCGs). The results of CCK-8 showed that the overexpression of CircTHBS1 promoted the viability of pCGs, while the deletion of CircTHBS1 reduced the cell viability. Knocking out CircTHBS1 also inhibited the migration of pCGs. In addition, we demonstrated that CircTHBS1 played a role in the adsorption of miR-211 by “sponge” in pCG. In turn, miR-211 can directly target CYCLIN D2 (CCND2) 3′UTR to perform its function. Finally, we confirmed the role and mechanism of CircTHBS1/miR-211/CCND2 regulation axis in pCGs. In summary, our study is the first to reveal the role and underlying mechanism of CircTHBS1 in CG, providing a potential biomarker and therapeutic target for human CG.

## Introduction

Cystitis glandular (CG) is a proliferative lesion of the bladder mucosa [1–3]. In recent years, the incidence of cystitis glandular has been increasing year by year. Its clinical symptoms are non-specific, mainly manifested as recurrent urinary frequency, urgency, pain, dysuria and microscopic or gross hematuria, pain and discomfort in the lower abdomen pelvic cavity, and mucus discharge in some patients [4]. Therefore, it is important to study the pathogenesis of glandular cystitis [5].

CircRNA is a class of non-coding RNA molecules that covalently combine at the 3′ and 5′ ends to form a closed loop. CircRNA is widely present in eukaryotic cells such as plants, animals, and humans. CircRNA, which is widely expressed in mammalian cells, has tissue-cell specificity, structural stability, and sequence conservation. Studies have shown that circRNA is involved in the transcription and expression of genes through many pathways and play an important role in physiological processes such as cell cycle or aging. More and more studies show that circRNA plays an important role in the occurrence and development of diseases including inflammation and cancer. MicroRNA (miRNA) is a type of single-stranded non-coding small-molecule RNA with a length of 21–23 nucleotides [6]. It regulates gene expression at the post-transcriptional level, thereby realizing the regulation of physiological and pathological processes such as tissue development, individual development, and tumorigenesis. Recent studies have found that microRNA-211 (miR-211) is closely related to the occurrence and development of various diseases, may play a role through multiple molecular biological pathways, and may affect high mobility gene groups related to gender determination. The expression of a variety of cell functional proteins such as SOX4 achieves therapeutic effects and is expected to become a therapeutic target for various diseases.

The gene encoding cyclinD2 is located at 12p13 and is called cyclin D2 (CCND2) [7]. The expression of CCND2 fluctuates in normal diploid cells and Rb-positive tumor cells, and its peak is at the late stage of G1. Injecting a small amount of CCND2 antibody into G1 phase cells can cause lymphocytes expressing CCND2 to stagnate in G1 phase, indicating that CCND2 is necessary for cells to metastasize from G1 phase to S phase and participates in various physiological and pathological processes [8,9].

In the present study, we aimed to investigate the role and mechanism of circular RNA THBS1 in primary cells of CG and adjacent normal tissues. We found that circTHBS1 promotes the viability and migration of pCGs by regulating the miR-211/CCND2 regulatory axis. This study demonstrated the role and potential mechanisms of circTHBS1 in CG and provided a potential direction and therapeutic target for CG.

## Methods

### Human specimen collection

The present study was approved and monitored by the Hospital Research Ethics Committee of Mianyang Central Hospital. Glandular cystitis and normal specimens were collected from patients undergoing surgical resection at the hospital. All patients obtained informed consent.

### Cell isolation and culture

The surgically obtained specimens immediately separated primary cells from glandular cystitis and normal controls. The cells were grown in Dulbecco’s modified Eagle’s medium (DMEM; Invitrogen, Carlsbad, U.S.A.). The cell culture medium contains 10% fetal bovine serum (FBS, Invitrogen), 100 U/ml penicillin, and 100 U/ml streptomycin (Invitrogen). All cell lines were cultured in a cell incubator (Thermo Scientific, MA, U.S.A.) at 37°C, 95% air, and 5% CO_2_.

### Cell transfection

CircTHBSA1, miR-211, and CCND2 siRNAs and mimic were purchased from Shanghai Gima. Transfection was performed in six-well plates, and plasmids (2 μg) or RNA oligonucleotides (50 μm) were transfected into each well. According to the instructions, 5 μl of Lipofectamine® 3000 (Invitrogen) transfection reagent was used. After transfection, after 6 h in culture, fresh medium containing 10% fetal bovine serum was used instead of medium without fetal bovine serum. These cells were then cultured for further research.

### Transwell assay detects cell migration capacity

After transfection, 1 × 10^5^ cells in logarithmic phase were resuspended in 150 μl serum-free medium and added to the upper chamber of the Transwell chamber. Add 500 μl of serum-containing complete medium to the Transwell lower chamber. The cells were cultured in a cell culture incubator at 37°C for 48 h. The upper chamber culture medium was discarded, the upper membrane was removed, and the non-membrane penetrating cells were removed. Cells were fixed in methanol for 15 min, and washed with PBS three times. Stained with 1% Crystal Violet for 20 min, and washed three times with PBS. Remove the bottom membrane of the upper chamber, observe, and count the cells passing through the small hole under a light microscope. Transwell Cell was purchased from Corning Corporation.

### Double luciferase reporter assay

The luciferase experiments are grouped according to the experimental purpose. The cells were seeded in a 24-well plate at a density of 1 × 10^5^ per well. When the cells were approximately 70% confluent the next day, 3′UTR luciferase plasmids containing CircTHBS1 or CCND2 were co-transfected with miR-211 mimicry. The substance or control was routinely cultured for 48 h, and then measured and analyzed on a multifunctional luminance meter according to the instructions of the dual luciferase reporter gene detection kit. Dual luciferase reporter gene kit and reporter vector were purchased from Promega.

### Cell viability assay

After the cells were cultured normally, they were pretreated by experimental grouping. They plant in 96-well plates. Five duplicate wells were designed. The detection time was 48 h. The detection reagent was applied with CCK8. After 2 h of normal incubation, the absorbance (450 nm) was measured for data analysis, and cell proliferation was detected.

### Detection of mRNA expression in tissues and cells by PCR

Clinical samples and cells were collected 48 h after transfection, and total RNA was extracted from tissues and cultured cells using TRIzol one-step method. Subsequently, DNase was used to process and 1 μg of RNA was reverse-transcribed to prepare cDNA. Then, 2 μl of the reverse transcription product was used for PCR detection. U6 and GAPDH were used as internal controls. The primer sequences were as follows: GAPDH, F: 5′-GGTGAAGGTCGGAGTCAACG-3′, R: 5′-CAAAGTTGTCATGGATGHACC-3′; CCND2, F: 5′-GCAGAACCTGTTGACCATCG-3′, R: 5′-GCTTGCGAAGGATGTGCTC-3′. Then, set up a PCR reaction system with a final volume of 20 μl, 2 μl of reverse transcription product, 10 μl of SYBR Green Mix, and 0.5 μl of upstream and downstream primers (10 μmol/l) according to the kit instructions. The parameters of PCR thermal cycling are: 95°C for 5 min, and then three steps of reaction: denaturation at 94°C for 30 s, annealing at 60°C for 30 s, a total of 45 cycles. The test results were calculated using the 2-ΔΔ*C*_t_ method.

### Statistical methods

Data analysis was performed using SPSS 22.0 software. Data with normal distribution between the two groups were tested using the *t* test, while single-factor ANOVA analysis was used for more groups. The difference was statistically significant with *P*<0.05.

## Results

### CircTHBS1 promotes cell viability and migration capacity of primary CG cells

**Figure 1:**
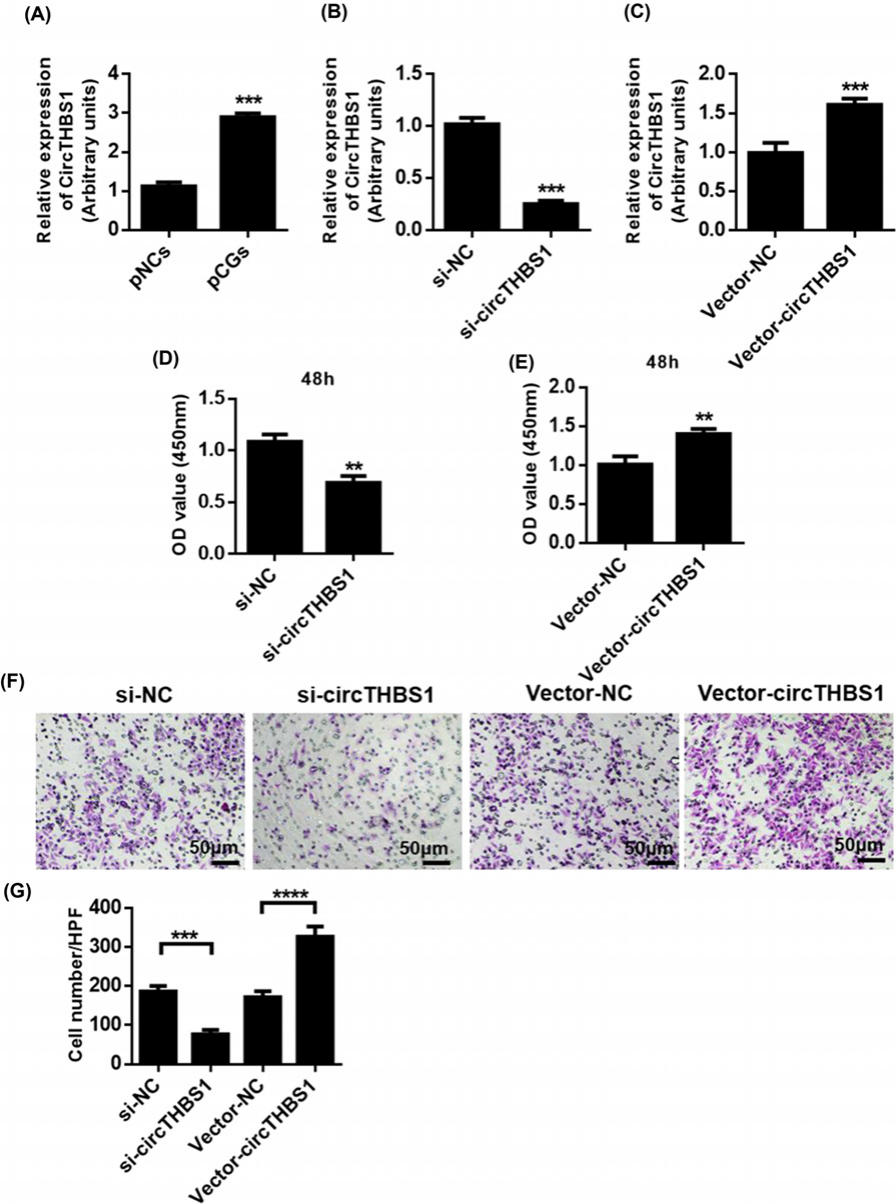
CircTHBS1 promotes cell viability and migration capacity of primary CG cells (**A**) QRT-PCR analysis of CircTHBS1 expression in pCGs and pNCs. (**B**) QRT-PCR analysis of the effectiveness and specificity of siRNA against CircTHBS1 in pCGs. (**C**) QRT-PCR analysis of the effectiveness of CircTHBS1 expression clone (Vector-CircTHBS1) in pCG. (**D**) CCK8 analysis to analyze the inhibition of cell viability in CircTHBS1 by pCG. (**E**) CCK8 analysis was used to analyze the viability of cells in pCG overexpressing CircTHBS1. (**F, G**) Transwell analyzed the cell migration potential of CircTHBS1 in which pCG was inhibited or overexpressed. **P<0.01, ***P<0.001, ****P<0.0001.

To study the role of CircTHBS1 in CG, we first isolated pCGs and pNCs cells. Experimental results show that CircTHBS1 is up-regulated in pCGs compared with pNCs (Figure 1A). For further research, we performed knockdown and overexpression of CircTHBS1, respectively. The experimental results show that siRNA can effectively reduce the expression of CircTHBS1 (Figure 1B), and the overexpression plasmid can also effectively increase the expression of CircTHBS1 (Figure 1C). Cell viability assay results showed that knockout of CircTHBS1 inhibited the viability of pCGs (Figure 1D), while overexpression of CircTHBS1 promoted the viability of pCGs (Figure 1E). Transwell analysis analyzed the cell migration potential of CircTHBS1, where pCG was inhibited or overexpressed. The results showed that knockout of CircTHBS1 inhibited the migration of pCGs, while overexpression of CircTHBS1 promoted the migration of pCGs (Figure 1F,G).

### CircTHBS1 adsorbs miR-211 through a “sponge”

**Figure 2:**
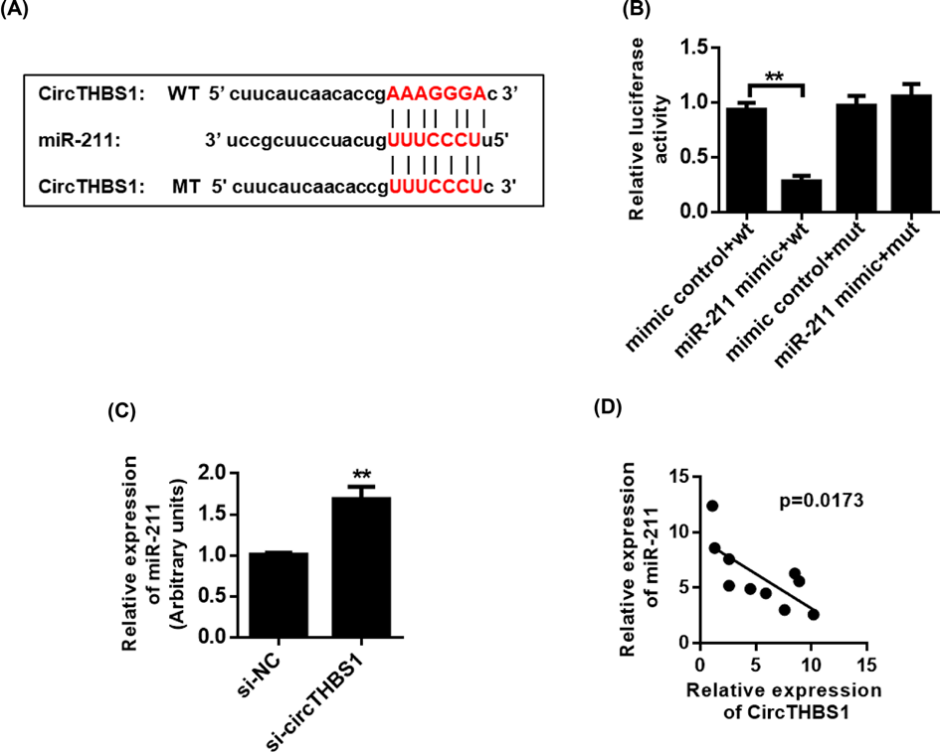
CircTHBS1 bind to miR-211 through a “sponge” (**A**) Schematic of the predicted binding sites of miR-211 and CircTHBS1. (**B**) Analysis of luciferase reporter gene binding between miR-211 and CircTHBS1. (**C**) qRT-PCR analysis of miR-211 expression. (**D**) Correlation analysis of co-expression of miR-211 and CircTHBS1. **P<0.01.

In order to study the target genes of CircTHBS1, we used Starbase (http://starbase.sysu.edu.cn/index.php) to predict the miRNAs it can bind. The experimental results show that CircTHBS1 can be bind to miR-211 (Figure 2A). Further dual luciferase reporter assays confirmed the binding of CircTHBS1 to miR-211 (Figure 2B). QRT-PCR results of miR-211 showed that knockout of CircTHBS1 could increase the expression of miR-211 (Figure 2C). Co-expression analysis of CircTHBS1 and miR-211 at the tissue level of patients showed that their expression was negatively correlated (Figure 2D).

### MiR-211 reverses the biological effects of CircTHBS1

**Figure 3:**
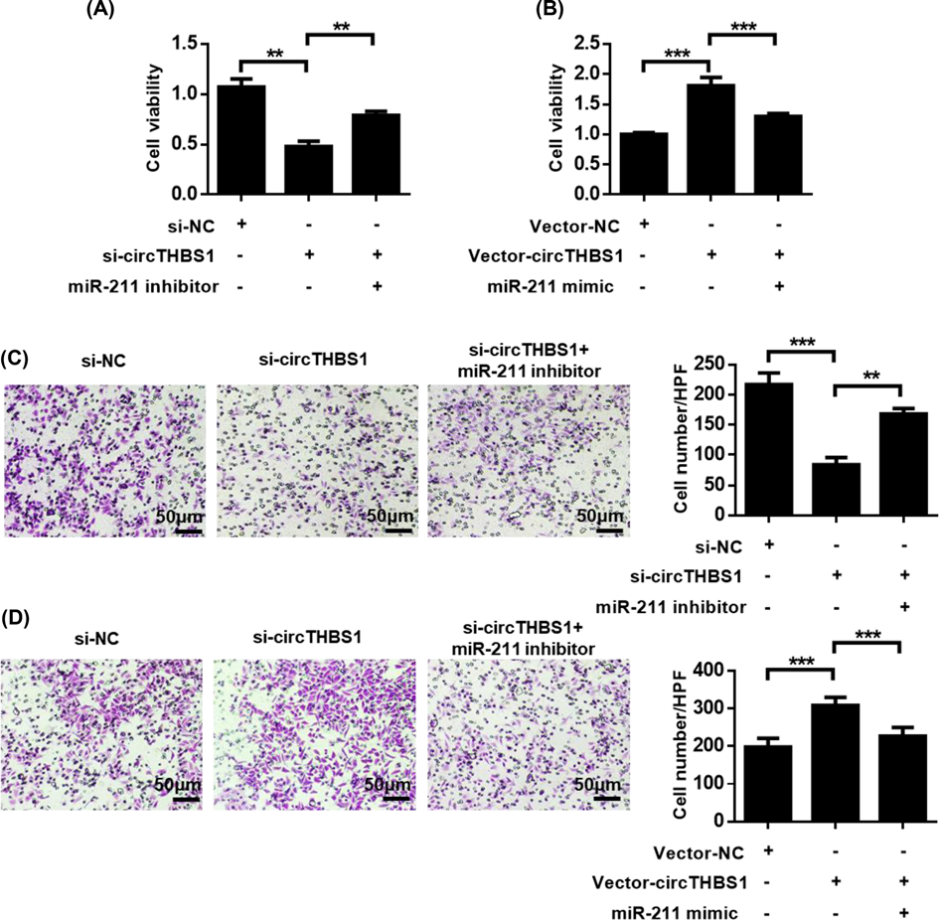
MiR-211 reverses the biological effects of CircTHBS1 (**A**) CCK8 analysis of pCG cell activity in siNC, siCircTHBS1, siCircTHBS1 + miR-211 inhibitor group. (**B**) CCK8 assay to analyze cell viability in pCG. (**C**) Transwell analysis of migration ability of pCG cells in siNC, siCircTHBS1, and siCircTHBS1 + miR-211 inhibitor group. (**D**) Transwell assay to analyze cell migration ability in pCG. **P<0.01, ***P<0.001.

We then used cell viability experiments and transwell experiments to elucidate whether miR-211 could affect the function of CircTHBS1 in primary CG cells. The analysis of cell viability experiments showed that miR-211 inhibitor inhibited miR-211 can reduce the inhibition of cell viability induced by si-circthbs1. At the same time, miR-211 overexpression can inhibit CircTHBS1-overexpression and induce cell viability (Figure 3A,B). Transwell analysis showed that miR-211 inhibitors reduced siCircTHBS1-induced cell migration potential, and overexpression of CircTHBS1 in pCGs cells restored miR-211 mimetic inhibitory migration potential (Figure 3C,D). The above results indicated that miR-211 reverses the function of CircTHBS1 in primary CG cells. Therefore, the role of CircTHBS1 in CG cells may be mediated by miR-211.

### MiR-211 regulates the expression of CCND2 in pCGs

**Figure 4:**
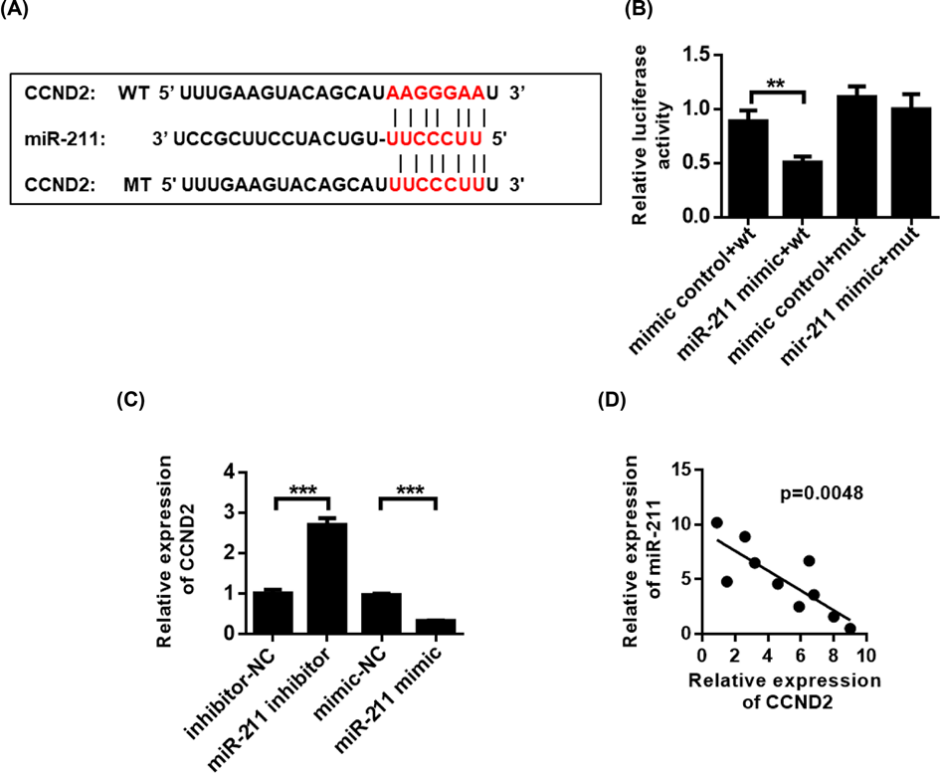
MiR-211 regulates CCND2 expression in pCG (**A**) Schematic diagram of the binding sites of miR-211 and CCND2. (**B**) The luciferase reporter analyzes the binding between miR-211 and CCND2 in pCG. (**C**) Analysis of CCND2 expression after miR-211 inhibition. (**D**) Correlation analysis of co-expression of miR-211 and CCND2. **P<0.01, ***P<0.001.

In order to analyze the target genes that miR-211 binds to, we use TargetScan to predict the target gene information of miR-211. The prediction results show that miR-211 may be bind to CCND2 (Figure 4A). Double luciferase reporter assays further confirmed that miR-211 can be bind to CCND2 (Figure 4B). CCND2 expression test results showed that inhibition of miR-211 can increase the expression of CCND2, while miR-211 inhibitor can reduce the expression of CCND2 (Figure 4C). The co-expression analysis of miR-211 and CCND2 showed that their expression was negatively correlated (Figure 4D).

### MiR-211 exerts its function by regulating CCND2 expression

**Figure 5:**
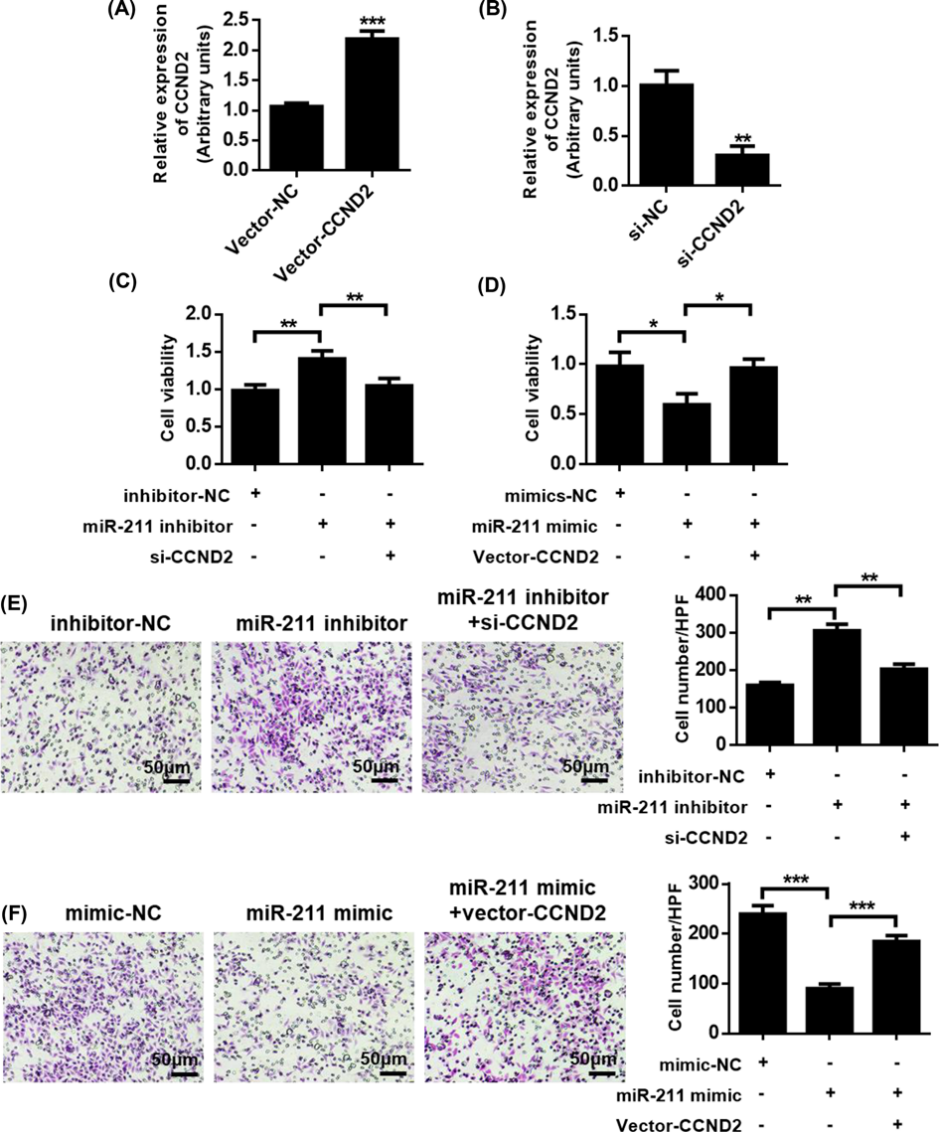
MiR-211 exerts its function by regulating CCND2 expression (**A**) qRT-PCR analysis of the effectiveness of CCND2 expression clones in pCG. (**B**) qRT-PCR analysis of the effectiveness and specificity of CCND2 siRNA in pCG. (**C**) CCK8 detects cell viability. (**D**) CCK8 detects cell viability. (**E**) Transwell assay for cell migration. (**F**) Transwell assay for cell migration. *P<0.05, **P<0.01, ***P<0.001.

Further, we investigated whether CCND2 could affect the role of miR-211 in primary CG cells. We first constructed the CCND2 overexpression plasmid and designed a specific siRNA against CCND2. QPCR analysis showed that CCND2 overexpressing clones significantly increased CCND2 expression, and siRNA also effectively reduced the expression of CCND2 (Figure 5A,B). The results showed that miR-211 inhibitors promoted cell viability and cell migration potential of primary CG cells. However, promotion induced by miR-211 was suppressed by siCCND2 (Figure 5C,D). In addition, CCND2 overexpression restored the miR-211 mimetic’s inhibitory effect on pCG cell migration potential (Figure 5E,F). These results demonstrate that miR-211 exerts its function by regulating the expression of CCND2 in primary CG cells.

### CircTHBS1 plays a role by regulating the CircTHBS1/miR-211/CCND2 axis

**Figure 6:**
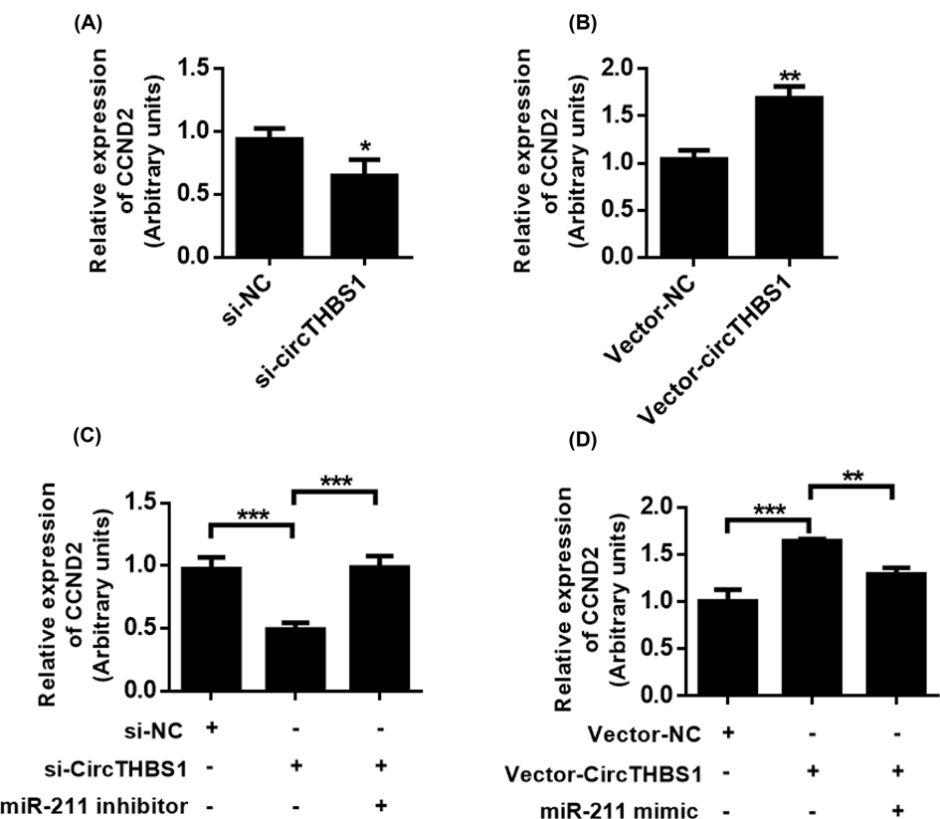
CCND2 is regulated by the CircTHBS1/miR-211 pathway (**A**) pCGs cells transfected with siCircTHBS1 and control siRNA to detect CCND2 expression. (**B**) pCGs cells transfected with vector-CircTHBS1 and control vector, and CCND2 expression was detected. (**C**) Detection of CCND2 expression. (**D**) Detection of CCND2 expression.

**Figure 7:**
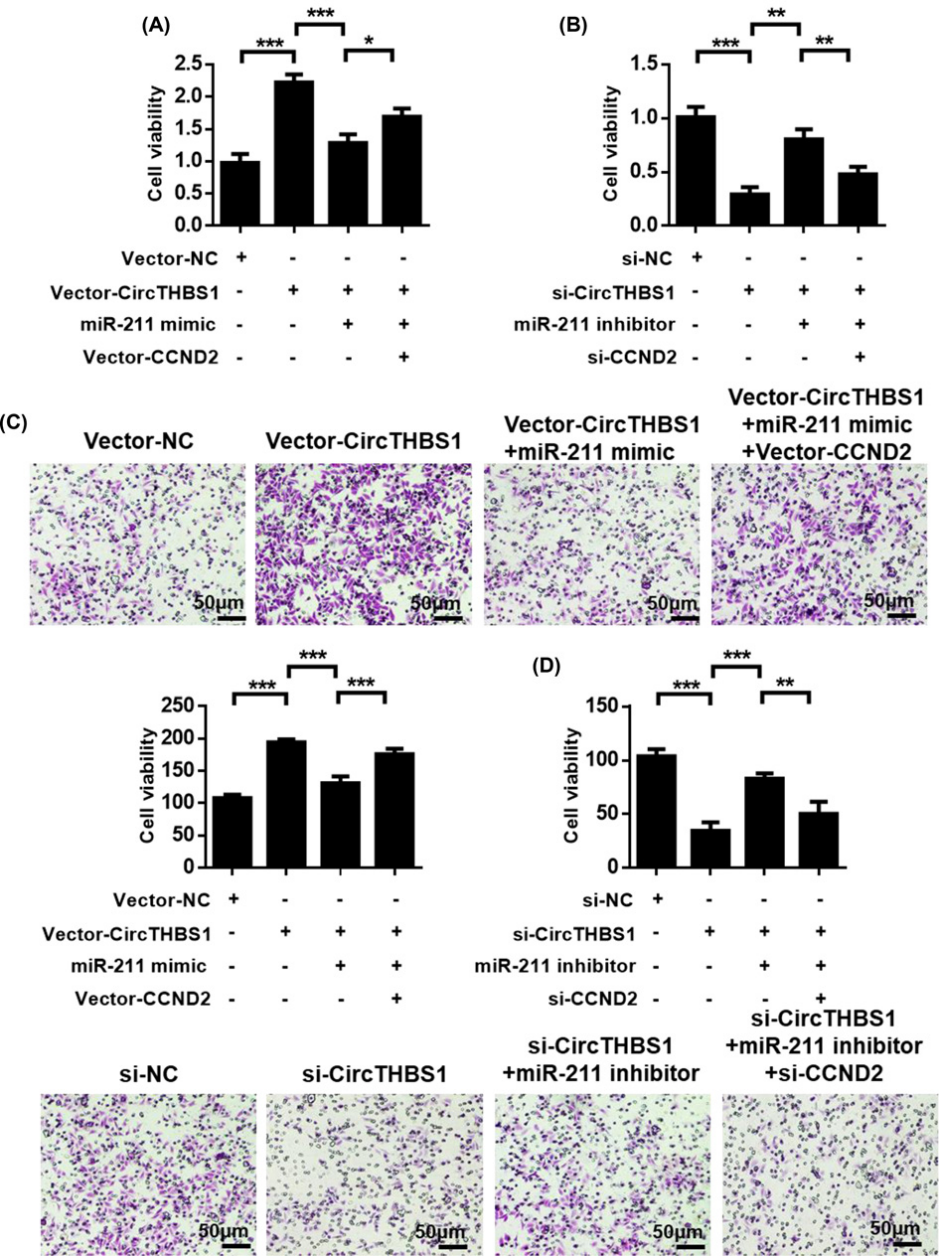
CircTHBS1 work by adjusting the CircTHBS1/miR-211/CCND2 axis (**A**) Cell viability in pCG was analyzed using different processing methods. (**B**) Cell viability in pCG was analyzed using different treatment methods. (**C**) Transwell analysis of cell migration in pCG using different processing methods. (**D**) Transwell analysis of cell migration in pCG using different processing methods.

We found that CircTHBS1 played a role in pCG by “sponge” adsorption of miR-211, while miR-211 played its role by regulating the expression of CCND2. Next, we investigated whether CircTHBS1 plays a role in primary CG cells via the miR-211/CCND2 axis. We first examined whether CircTHBS1 could regulate the expression of CCND2. QPCR analysis showed that knockdown of CircTHBS1 inhibited the expression of CCND2, while overexpression of CircTHBS1 up-regulated the expression of CCND2 (Figure 6A,B). Next, we investigated whether CCND2 was involved in the regulation of miR-211 by CircTHBS1. QPCR analysis showed that miR-211 inhibitor restored siCircTHBS1-induced CCND2 expression inhibition, while miR-211 mimic inhibited CCND2 expression increase in pCGs induced by cIRCTHBS1 overexpression (Figure 6C,D). Further, we investigated the function of the CircTHBS1/miR-211/CCND2 regulatory pathway in primary CG cells. Cell viability and migration analysis showed that the regulation of the CircTHBS1/miR-211/CCND2 regulatory pathway would affect the cell viability and migration potential of primary CG cells (Figure 7A,B). Together, these results suggest that CircTHBS1 plays a role by regulating the CircTHBS1/miR-211/CCND2 regulatory axis in primary CG cells (Figure 7C,D).

## Discussion

The etiology and pathogenesis of glandular cystitis are still controversial, but it is generally believed that glandular cystitis is a neoplastic lesion of urothelial hyperplasia formed in the role of long-term chronic stimulation [10–12]. Since the etiology and pathogenesis are unknown, there is no specific treatment plan [13,14]. Therefore, it is urgent to study the pathogenesis and therapeutic targets of glandular cystitis, so as to provide references for subsequent treatment [15,16].

It was found that circRNA contained miRNA-binding sites in non-coding transcripts or 3′-UTRs of specific genes [17]. CircRNAs can bind miRNA as a competitive inhibitor, inhibit the activity of related miRNA, and then affect gene expression [18]. The present study found that CircTHBS1 adsorbed miR-211 through a “sponge”. CircRNA plays an important role in urinary diseases. Zhong et al. [19] found that the expression of six circRNAs in bladder cancer tissue and normal tissue was statistically significant: circTCF25, circZFR, circPTK2, and circBC048201 were all up-regulated, while circFAM169A and circT-RIM24 were down-regulated. Overexpression of circTCF25 can down-regulate the activity of miR-103a-3p and miR-107, increase the expression of CDK6, and promote the proliferation and migration of bladder cancer cells. Huang et al. [20] found nine circRNAs with miRNA-binding sites. Because these target genes are involved in the development and progression of cancer cells, circRNA may be involved in the pathogenesis and development of bladder cancer. This circRNA–miRNA interaction is the bladder. Cancer mechanism provides a new perspective [21].

The main mechanism of action of microRNA is to form a complementary pairing with the non-coding region (3′-UTR) of the target protein’s mRNA, thereby degrading it and inhibiting the expression of the target protein [22]. MiR-211 is located on chromosome 15q13, which is closely related to tumorigenesis and development [23]. Although studies have shown that it is related to the occurrence, development, invasion, and metastasis of a variety of malignant tumors, the exact role of miR-211 in glandular cystitis has not been elucidated [24]. In the present study, miR-211 mimic were successfully used to transfect primary glandular cystitis cells, and then the exact inhibitory effect on migration ability was detected, indicating that miR-211 inhibited the invasion and migration of glandular cystitis cells. The present study further demonstrated that miR-211 can directly regulate CCND2 expression.

According to biological information tools, CCND2 (cyclinD2) is predicted to be a possible target gene of miR-211 [25,26]. CCND2 as a member of the cell cycle family, its abnormal expression may lead to abnormal cell proliferation. Studies have found that CCDN2 is abnormally expressed in a variety of tumor tissues, such as cervical cancer [27], gastric cancer [28], non-small cell lung cancer [29], and prostate cancer [30,31]. At the same time, HUANG et al. reported that miR-615 inhibits the proliferation and invasion of prostate cancer cells by targeted down-regulation of CCND2. In addition, miR-1297 [32], miR-154, and miR-497 [33] have been reported to target down-regulation of CCND2 to inhibit tumor cell proliferation and migration [34–37]. For this reason, the present study speculates and validates that miR-211 regulates the proliferation and invasion of glandular cystitis cells through targeted regulation of CCND2.

## Conclusion

In the present study, we demonstrated the role of CircTHBS1 in the cell viability, cell proliferation, and migration potential of pCGs, and revealed that CircTHBS1 exerts its function by regulating the miR-211/CCND2 pathway in pCGs. Therefore, our study provides a potential biomarker and therapeutic target for CG.

## Data Availability

No additional data are available.

## Abbreviations

CCND2: CYCLIN D2
CG: cystitis glandular
miR-211: microRNA-211
pCG: primary CG cell
